# Active Learning on Dynamic Clustering for Drift Compensation in an Electronic Nose System

**DOI:** 10.3390/s19163601

**Published:** 2019-08-19

**Authors:** Tao Liu, Dongqi Li, Jianjun Chen, Yanbing Chen, Tao Yang, Jianhua Cao

**Affiliations:** School of Microelectronics and Communication Engineering, Chongqing University, No. 174 Shazheng Street, Chongqing 400044, China

**Keywords:** active learning, drift counteraction, dynamic clustering, electronic nose

## Abstract

Drift correction is an important concern in Electronic noses (E-nose) for maintaining stable performance during continuous work. A large number of reports have been presented for dealing with E-nose drift through machine-learning approaches in the laboratory. In this study, we aim to counter the drift effect in more challenging situations in which the category information (labels) of the drifted samples is difficult or expensive to obtain. Thus, only a few of the drifted samples can be used for label querying. To solve this problem, we propose an innovative methodology based on Active Learning (AL) that selectively provides sample labels for drift correction. Moreover, we utilize a dynamic clustering process to balance the sample category for label querying. In the experimental section, we set up two E-nose drift scenarios—a long-term and a short-term scenario—to evaluate the performance of the proposed methodology. The results indicate that the proposed methodology is superior to the other state-of-art methods presented. Furthermore, the increasing tendencies of parameter sensitivity and accuracy are analyzed. In addition, the Label Efficiency Index (LEI) is adopted to measure the efficiency and labelling cost of the AL methods. The LEI values indicate that our proposed methodology exhibited better performance than the other presented AL methods in the online drift correction of E-noses.

## 1. Introduction

Olfactory perception based on Electronic noses (E-noses) [1] has attracted much attention in research communities in recent years. Although the feasibility of E-noses has been demonstrated with various solutions in a considerable number of cases, the drift problem still hampers its further development. Drift, normally caused by environmental and physicochemical factors, disturbs the compatibility between the gas-sensor responses and the Artificial Intelligence (AI) algorithms in E-nose systems. It is evident that gas-sensor drift would irreversibly mislead the AI models over time. In other words, drift will significantly influence the performance of E-nose systems that make decisions based on AI algorithms.

There are several ways to mitigate the negative effects of gas-sensor drift. The primary option is gas-sensor enhancement, which improves the sensor’s repeatability and stability by employing an advanced structure or film composition. Aiming for more universal and cost-effective methodologies, algorithm-based methods are becoming fashionable. Among these, a number of methods are used for decomposing the drift component from responses of the gas-sensor-array. Accordingly, what remains are the corrected responses after drift correction. In practice, the drift component can be decomposed based on statistical characteristics by means of Principal Component Analysis (PCA), Common PCA (CPCA) [2], PCA-based Component Correction (PCA-CC) [3], Independent Component Analysis (ICA) [4,5], and wavelet [6,7]. Additionally, it is possible to identify components unrelated to the classification as drift signals using Orthogonal Signal Correction (OSC) [8,9], Partial Least Squares (PLS) [10], and Linear Discriminate Analysis (LDA) [11]. Furthermore, machine-learning approaches can be used to correct the recognition models, instead of the gas-sensor responses. Adaptive classifiers, including Self-Organizing Maps (SOM) [12,13] and Adaptive Resonance Theory (ART) [14,15], have been adopted as recognition units that train themselves automatically with drifted gas samples. Moreover, some researchers have assumed that current drift is related to previous ones; accordingly, the recognition output of a drifted sample can be expressed as an output-ensemble of several sub-classifiers [16,17,18,19,20] that have been trained on drifted gas samples during different periods. Thus, the final output is one that has been corrected based on previous drift tendencies. We should note that sufficient correction samples, full and complete categories, and sufficient correction time are essential prerequisites of all of the above methods. Unfortunately, these requirements may not be fully satisfied in many practical uses, e.g., there may be insufficient time to collect enough samples, or it may be too expensive to obtain the categories of the correction samples.

To extend the possible applications of E-noses, we divided a conventional drifted sample for drift correction into two parts: the instance and the label. Here, “instance” refers to the feature vector abstracted from the gas-sensor-array responses in one experiment, while “label” refers to the category of a “sample”. In real applications, the two parts are not always presented together, and the “label” may be costly to access. Hence, new machine-learning paradigms have been adopted to deal with this challenge. Luo et al. utilized a deep-learning neural network to abstract the drift-independent features for recognition in [21], eliminating the drift of the features. Zhang et al. focused on the situation in which the labels for the drifted instances are unknown [11,22]. Meanwhile, Liu et al. presented a label estimation method with manifold learning and joint kernels to achieve pseudo-labels for drift correction [23]. Considering online situations in which there are many drifted instances and only a small number of labels are accessible (due to the high cost of labelling), Domain Adaptation Extreme Learning Machines (DAELM) [24], Standardization Error-based Model Improvement (SEMI) [25], and Transfer sample-based Coupled Task Learning (TCTL) [26] have been proposed to maximize the information-usage efficiency of both instances and labels. However, these methodologies designate samples for drift correction randomly or subjectively, making the anti-drift effect unstable or non-convergent. Therefore, Active Learning (AL) was introduced for drift correction, aiming to tag labels to limited instances selectively and intentionally [27].

According to previous study, AL has already been used to improve the classification precision of E-noses [28], as well as being employed as a suitable framework for online drift compensation (many drifted instances and limited labels). The core of the AL framework is its “instance-selection strategy”, which filters out the instances needing labelling. At present, stream-based [29] and pool-based [30] ALs are popular instance-selection strategies. The stream-based strategy chooses instances based on a fixed threshold that relies on local data information, whereas the pool-based strategy selects appropriate instances based on the global data distribution. Furthermore, the pool-based strategy can be divided into several sub-types. Uncertainty Sampling (US) [31], Query-By-Committee (QBC) [32], and Error Reduction (ER) sampling [33] are typical among pool-based strategies.

In this paper, we propose an advanced AL methodology for Dynamic Clustering (AL-DC) in order to improve the effect of drift correction under online conditions. For AL-DC, drifted instances are collected together in a data pool and automatically allocated to several non-overlapping clusters. Then, the instance-selection process alternately selects instances from each cluster. In other words, we create an AL mechanism that makes the category ratio of the selected instances as equal as possible. A public database was adopted to verify the effectiveness and robustness of the proposed method. Based on the experimental results, AL-DC achieved higher accuracy than any of the other state-of-the-art methodologies in a practical online scenario. AL-DC also demonstrated faster accuracy convergence than the other AL methods. Additionally, we used the Labelling Efficiency Index (LEI) to comprehensively measure the implementation efficiency and the labelling cost.

The rest of the paper is structured as follows: we detail the traditional AL and the proposed AL-DC methods in Section 2. Section 3 describes the E-nose drift database used in both the long-term drift and short-term drift scenarios. In Section 4, we present and discuss the results achieved using the database. Finally, we summarize our conclusions in Section 5.

## 2. Methods

### 2.1. Basic Active Learning Framework

**Figure 1:**
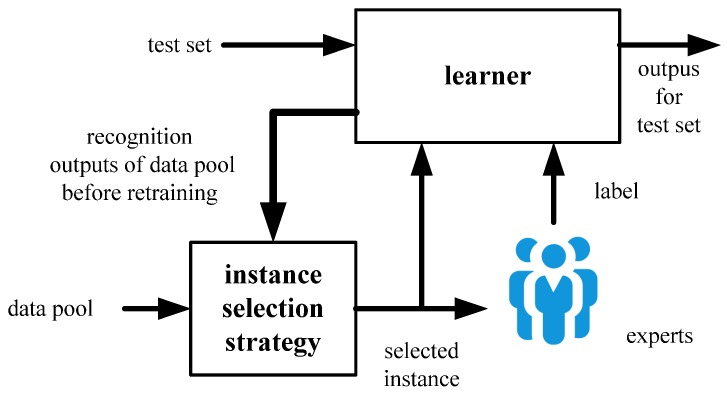
Diagram of active learning.

The framework of the AL process is presented in Figure 1. It is an obvious closed-loop structure, which retrains the “learner” iteratively by the “selected instance” and its “label”. The “selected instance” is chosen from the “data pool” full of drifted instances, while the “label” to the “selected instance” is queried from the “experts”. The “instance selection strategy” regularly determines the instance near the classification boundary with a certain rule for learner retraining. The “experts” implement manual labelling to provide a label to the selected instance. Finally, the “learner” is renewed with the selected instance and the label for next-round instance selection.

For online drift correction, we often face the problem whereby continuous gas instances are easy to achieve, but their labels are rare. Thus, “selecting a limited number of gas instances—querying associated labels from experts” becomes an effective approach for forming samples for online drift correction. AL is a feasible method for guiding optimal instance selection. It intends to achieve higher learner performance with fewer human annotations.

### 2.2. Basic Instance-Selection Strategy 

AL regards manual labelling as the most time-consuming and labor-intensive part. Therefore, instance-selection strategies that explore the most useful instance for learner correction are the key part of the AL paradigm. Considering the benefits of pool-based instance selection, we would like to introduce three pool-based instance-selection strategies (US, QBC, and ER) in the following subsections.

#### 2.2.1. Uncertainty Sampling

The US strategy explores the instance that makes the greatest contribution in terms of classifier updating according to the outputs of the recognition component. Various metrics can be adopted to indicate the uncertainty of the instance based on the recognition outputs. In this study, a popular metric named margin [32] is chosen, as follows:

(1) $$margin_{i}=f^{E}({\hat{y}}_{c1}|p_{i})-f^{E}({\hat{y}}_{c2}|p_{i}),$$

where *margin_i_* denotes the margin value of the *i*-th instance *p_i_*, $f^{E}({\hat{y}}_{c1}|p_{i})$ and $f^{E}({\hat{y}}_{c2}|p_{i})$ are, respectively, the maximum and second maximum posteriori probabilities of *p_i_* calculated by a classifier. According to this criterion, smaller *margin_i_* means greater uncertainty of *p_i_*. Thus, the margin-based US strategy is prone to selecting the instance with the minimum margin from a bunch of instances.

#### 2.2.2. Query by Committee

In contrast to US, QBC uses multiple classifiers to perform instance recognition and evaluation. Typically, one classifier (the chair) holds incoming instance recognition; another two classifiers (members) calculate the valuableness of the instances for human annotation. QBC identifies the instance most in need of annotation based on the disagreement of the two member outputs. Conventionally, Kullback–Leibler distance (KLD) [27] is the metric of disagreement, as follows:

(2) $$KL_{i}=\frac{1}{K}\sum_{k=1}^{K}D(P_{k}(C|p_{i})||\frac{\sum_{k}P_{k}(C|p_{i})}{K}),$$

(3) $$D(P_{k}(C|p_{i})||\frac{\sum_{k}P_{k}(C|p_{i})}{K}=\sum_{m=1}^{\left|C\right|}P_{k}(c_{m}|p_{i})\log\frac{K\cdot P_{k}(c_{m}|p_{i})}{\sum_{k}P_{k}(c_{m}|p_{i})},$$

where $KL_{i}$ is the KLD of the *i*-th sample *p_i_*. *K* denotes the total number of committee members. $P_{k}(c_{m}|p_{i})$ represents the probability that sample *p_i_* belongs to Class *c_m_* by Member *k*.

#### 2.2.3. Error Reduction Sampling

ER sampling prefers to select the instances that significantly minimize the generalization errors of learners. The process of ER sampling includes: (1) choose a loss function ${\tilde{E}}_{{\hat{P}}_{D^{*}}}$ to estimate the generalization errors; (2) estimate each instance with the loss function; (3) select an instance for labelling with the largest reduction of loss-function values. In the ER sampling process, the form of the loss function has a great impact on the performance. Both logarithmic loss and 0/1 loss functions are common criteria, as follows:

(4) $${\tilde{E}}_{{\hat{P}}_{D^{*}}}=\frac{1}{\left|P\right|}\sum_{x\in P}\sum_{y\in Y}{\hat{P}}_{D^{*}}\left(y|x\right)\log\left(\frac{1}{{\hat{P}}_{D^{*}}\left(y|x\right)}\right),$$

(5) $${\tilde{E}}_{{\hat{P}}_{D^{*}}}=\frac{1}{\left|P\right|}\sum_{x\in P}\left(1-\underset{y\in Y}{\max}{\hat{P}}_{D^{*}}\left(y|x\right)\right),$$

where ${\hat{P}}_{D^{*}}\left(y|x\right)$ is the posterior probability of Class *y* by a given sample *x*. *P* denotes the renewed data pool and *Y* is the category label set. In the following sections, we set the default loss function of ER as logarithmic loss.

### 2.3. Adaptive Confidence Rule

The Adaptive Confidence Rule (ACR) is a pool-based approach designed specifically for online drift correction of E-noses [27]. This method assumes that the entire data distribution of the drifted responses/features is continuously moving over time. Consequently, the instances near both classification planes and the category center are equally important for collection of drift information.

In the ACR procedure, instance evaluation is performed according to Equations (2) and (3), as in QBC. The difference between ACR and other AL paradigms lies in the rule of instance selection. ACR tries to record the outputs of the chair before and after classifier updating in each instance-selection process. If the outputs are different, it means the classification plane of the learner is not well trained. Therefore, the instance with the greatest KLD should be selected for learner retraining to distinguish the classification boundary of the drifted instances. In contrast, low KLD means the corresponding instance is near the center area of a certain category. Thus, ACR prefers to choose the instance with the lowest KLD when the two outputs from the chair are equal. This behavior would supply complete drift information in all domains for drift correction. Finally, ACR improves the representativeness of the selected instances under drifted data distribution.

### 2.4. Proposed Active Learning Strategy

**Figure 2:**
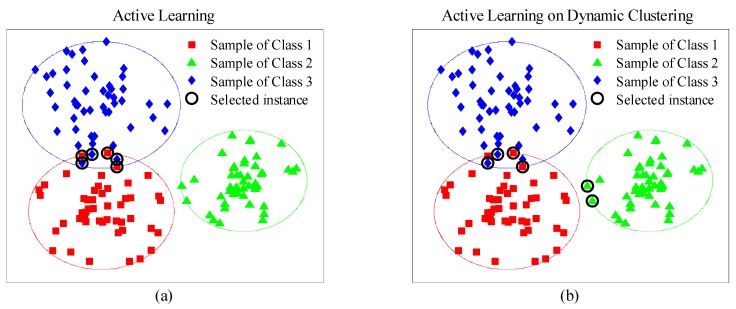
(**a**) Effects of traditional active learning; (**b**) effects of proposed AL-DC.

Traditional pool-based AL methods prefer to assess instances based on the instance distribution of the data pool. Accordingly, the selected instances may distribute on a local area (e.g., classification boundary of certain category). In other words, drift information of other domains (e.g., drifted instances of other categories) are barely to collect in AL process. Figure 2a demonstrates the common selected-instance distribution of the AL methods. We can see that all the selected instances are concentrated on the overlap between Class 1 and 3, and no instance emerges in the area of Class 2, because the boundary uncertainty of Class 2 is lower than those of Classes 1 and 3. Consequently, Class 2 cannot be corrected when the learner is retrained, which decreases the recognition accuracy of Class 2 dramatically. As shown in Figure 2b, our proposed methodology aims to pick instances considering both category and uncertainty. There are three stages of the proposed methodology AL-DC: initialization, clustering and instance selection.

#### 2.4.1. Initialization

Suppose a learner has initially been established by the training set with all kinds of samples. Then, AL-DC computes the initial means of all the samples in the training set by category:

(6) $${\bar{m}}_{k}^{s}=\frac{1}{N_{k}}\sum_{x_{i}^{s}\in c_{k}}x_{i}^{s},$$

where $x_{i}^{s}$ represents the *i*-th samples belong to Category *c_k_*. $N_{k}$ and ${\bar{m}}_{k}^{s}$ are the sample number and mean value of Category *c_k_*, respectively.

#### 2.4.2. Clustering

We define clustering mean ${\bar{m}}_{k}^{t}$ and let ${\bar{m}}_{k}^{t}={\bar{m}}_{k}^{s}$. Then, we perform clustering process as follows:

(7) $$c_{i}^{t}=\underset{k}{\arg}\min\left\|x_{i}^{t}-{\bar{m}}_{k}^{t}\right\|,$$

where $x_{i}^{t}$ and $c_{i}^{t}$ represent the *i*-th instance of the data pool and associated cluster, respectively. Afterwards, we update ${\bar{m}}_{k}^{t}$ by:

(8) $${\bar{m}}_{k}^{t}=\frac{1}{{N^{\prime }}_{k}}\sum_{x_{i}^{t}\in c_{i}^{t}}x_{i}^{t},$$

where ${N^{\prime }}_{k}$ is the number of instances belonging to Cluster *k* in the data pool. Then, we perform (7) and (8) iteratively until each ${\bar{m}}_{k}^{t}$ has converged.

Through the above steps in this stage, several sample clusters are obtained. We assume the samples in the same cluster have similar location and category. Meanwhile, the number of clusters is consciously equal to the number of categories, which ensures a distribution of clusters close to that of categories.

#### 2.4.3. Instance Selection

We define a binary flag vector $f=\left\{f_{1},f_{2},\cdots ,f_{K}\right\}$ and set all elements of **f** to 1, where *K* denotes the number of clusters. Then, we select the most valuable cluster by:

(9) $$k^{*}=\underset{k}{\arg}\max\{\frac{f_{1}}{{N^{\prime }}_{1}}\sum_{x_{i}^{t}\in c_{1}^{t}}I\left(x_{i}^{t}\right),\frac{f_{2}}{{N^{\prime }}_{2}}\sum_{x_{i}^{t}\in c_{2}^{t}}I\left(x_{i}^{t}\right),\cdots ,\frac{f_{K}}{{N^{\prime }}_{K}}\sum_{x_{i}^{t}\in c_{K}^{t}}I\left(x_{i}^{t}\right)\},$$

where I(·)>0 is the function that calculates the uncertainty value of a certain sample. k^*^ means the serial number of the most valuable cluster.

We set $f_{k^{*}}=0$ to avoid successive selecting in the same cluster. Then, we pick up the finest instance x^*^ in the most valuable cluster according to:

(10) $$x^{*}=\underset{x\in x_{k*}^{t}}{\arg}\max\{I\left(x\right)\},$$

Next, move x^*^ from the data pool to the training set and mark x^*^ with the label querying from the experts. During the repetition of Equations (9) and (10), we reset all the elements of **f** as 1 if $f_{1}=f_{2}=,\cdots ,=f_{K}=0$. In other words, the instances should be selected from each cluster alternately to maintain the balance of sample category of the training set. Moreover, Equations (10) makes larger informative (uncertainty) instances preferred in the selection, which helps the AL-DC methodology to converge faster on accuracy with the same labelling times. Finally, the whole methodology will stop when enough instances have been chosen, or certain precision has been satisfied. The details of AL-DC are summarized in Algorithm 1.

**Algorithm1.** Algorithm of active learning on dynamic clustering.
**Input:**
 The learner. The training set. The flag vector $f=\left\{f_{1},f_{2},\cdots ,f_{K}\right\}=\left\{1,1,\cdots ,1\right\}$.
**Output:**
  The renewed learner.
**Procedure:**

Compute the mean value ${\bar{m}}_{k}^{s}$ of each class based on (6);Cluster the instances in data pool according to ${\bar{m}}_{k}^{s}$ by (7) and (8) iteratively;Select instance x^*^ iteratively by (9) and (10);Delete x^*^ from data pool and add it to the training set;Renew the learner with the updated training set;Reset **f** if $f=\left\{f_{1},f_{2},\cdots ,f_{K}\right\}=\left\{0,0,\cdots ,0\right\}$;End if certain conditions are reached, otherwise, return to step 1.


## 3. Data and Settings

### 3.1. System and Dataset

The drifted data were generated from an E-nose system designed by the machine learning repository at UC Irvine [16]. For gas sensing, four commercial gas-sensor models (TGS2600, TGS2602, TGS2610, and TGS2620) were selected to form a 16-sensor array, that is, four sensors were employed of each model. During each experiment, the gas-sensor array was working at a constant temperature of 400 °C. The flow rates of the injected gases can be adjusted via three Mass Flow Controllers (MFCs), and the total flow rate through the gas chamber is kept at 200 mL/min.

There were six volatile compounds (ammonia, acetaldehyde, acetone, ethylene, ethanol, and toluene) adopted to the experiments over 36 months. A series of experiments was performed, and 13,910 samples were recorded to form a drift dataset. For ease of access, as shown in Table 1, all of the recorded data were arranged into 10 batches based on recording time. For each gas sensor, eight features were abstracted from the original response in one experiment. Among these features, two of them, namely, ΔR and its normalized version, indicated steady-state characteristics. The other six features were dynamic ones, called exponential moving average (ema*_a_*), abstracted from both the rising and decaying stages of the raw sensor responses. The ema*_a_* is calculated as follows:

(11) y[k]=(1−a)y[k−1]+a(r[k]−r[k−1]),

where *k* is a natural number indexing the discrete time at which the chemical vapor is present, *r*[*k*] is the time profile of sensor resistance and set *y*[0] = 0 initially. Three different values are set for *a* (*a* = 0.1, *a* = 0.01 and *a* = 0.001). Thus, three of six dynamic features were for the rising stage, and the others were for the decaying stage. Considering 16 gas sensors have been tagged on the gas-sensor array, each sample can finally be mapped into a 128-dimensional feature vector (8 features × 16 sensors).

**Figure 3:**
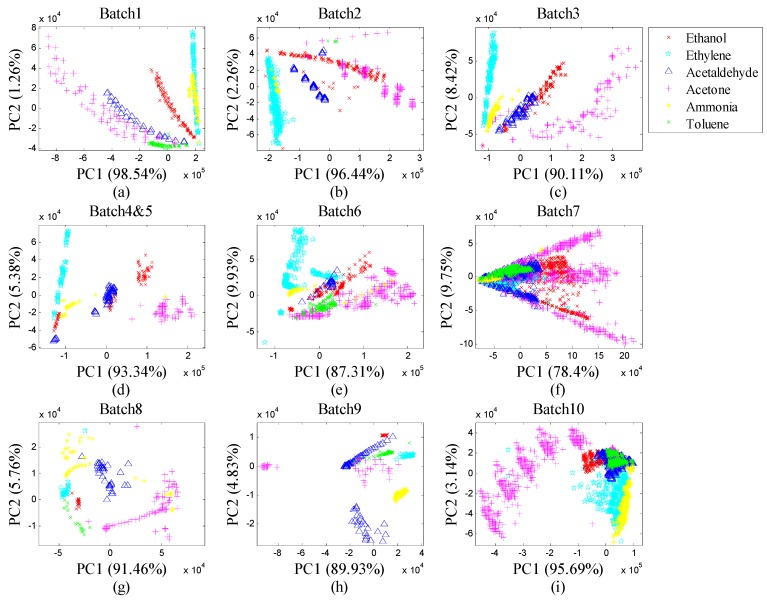
(**a**) Data distribution of Batch 1; (**b**) data distribution of Batch 2; (**c**) data distribution of Batch 3; (**d**) data distribution of Batch 4&5; (**e**) data distribution of Batch 6; (**f**) data distribution of Batch 7; (**g**) data distribution of Batch 8; (**h**) data distribution of Batch 9; (**i**) data distribution of Batch 10.

Considering the high-dimension of the raw feature vector, we utilized Principal Component Analysis (PCA) plots to visualize the distributions of the drifted samples by batch in Figure 3a–i. The gas samples with the same chemical composition were distributed differently from Batch 1–10, which implies the in-deed drift effects on the samples with time. This dataset is often used to evaluate drift correction methods that recognizes gas samples by compounds. In this paper, we used these 10 batches of data as continuous on-line drifted samples.

### 3.2. Experimental Setup

In the experiments, we arranged two E-nose drift scenarios with different settings:

“Setting 1” defines a long-term drift compensation scenario, which appoints Batch 1 as the initial training set and all the following batches are devoted to instance selection and testing. Each instance-selection process would be triggered when a new batch comes.

“Setting 2” creates short-term scenarios for drift counteraction, which uses two consecutive batches. The former batch is seen as the initial training set, while the latter one is used for instance selection and performance evaluation.

In terms of the learner, two classifier types, including Extreme Learning Machine (ELM) and Support Vector Machine (SVM), are adopted. For ELM, we used the Radial Basis Function (RBF) kernel and set the kernel parameters to 0.005. Considering both recognition rate and computational cost, we chose the linear kernel for SVM instead of the RBF kernel. Additionally, the penalty factor *C* of SVM was optimized in the range 10^−3^ to 10^3^ with a variable step size and set to 0.2.

To prove the superiority of the proposed methodology, we conducted the assessments in four stages. Primarily, we compared the proposed AL-DC methodology with other state-of-the-art methods with respect to recognition rate under drift effects. Higher recognition rate indicates better drift correction. Then, we discussed the parameter sensitivity of AL-DC with a varied selected-instances number. The methodology is easier to apply if it has lower parameter sensitivity. Thirdly, we explored the reason AL-DC achieves excellent performance. Finally, labelling efficiency of AL-DC is analyzed using the index *LEI*. Higher *LEI* signifies faster accuracy increase with the same time and labor costs. For the implementation of AL methods, we divided the data into initial training samples, an online data pool and a test set. We collected the initial training samples from the first batch for learner building. Each subsequent batch contains an online data pool and a test set. The online data pool was a set of drifted instances with no labels, which stored the candidates for labelling. After the learner was updated by the selected instances with labels, the remaining data were used for testing. Therefore, the learner can be retrained at each time a new batch arrives. Totally, 9 AL processes would be trigged for drift correction with either Setting 1 or 2. To be consistent with the setting of the comparison methods, the scope of data pool covered the whole batch in the first discussion stage. In other discussion stages, the ratio of data pool to test set was about 1:2. The detailed instance numbers of the data pool and testing sets are recorded in Table 2. For each batch, learner updating on AL was executed before learner testing.

## 4. Results and Discussion

### 4.1. Computational Environment 

The drift dataset was preserved in 10 txt files. We imported the dataset and established the proposed methodology model in MATLAB (2014a). The computation was executed on a desktop computer with the following configuration:

System: 64-bit, Windows 10.

Processor: Intel i5-8500.

RAM: 16 GB.

Hard disk: solid state disk 128GB.

### 4.2. Accuracy Comparison 

In this subsection, we employ several state-of-art drift counteraction methods for comparison. Four drift-correction types are presented: Component Correction (CC), Instance Correction (IC), Label-Free Correction (LFC) and AL. For CC, we chose two different approaches. CC-LDA [11] and CC-OSC [26] use LDA and OSC, respectively, to decompose the drift component from features and adopt SVMs as classifiers. TCTL+SEMI [26] and DAELM [24] are the representatives of the IC type, which renews the recognition models with drifted samples periodically and aimlessly. Moreover, the LFC type, which includes SVM-comgfk [23], ML-comgfk [23], the Multi-Feature Kernel Semi-supervised joint learning model (MFKS) [22], and Domain Regularized Component Analysis (DRCA) [11], assists with classifier updating in a no-label way, that is, the drift correction is performed on the drifted instances only. The last type is AL including AL-ACR [27] and our proposed AL-DC. Considering AL-DC is an open framework for an instance-selection strategy, we apply US, QBC and ER to AL-DC, which we denote as AL-DC-US, AL-DC-QBC and AL-DC-ER, respectively. For the methods which need labels, we add a number in brackets to indicate the sample size for drift compensation.

#### 4.2.1. Long-Term Drift

Table 3 demonstrates the accuracy of the methods under the long-term scenario. We compute the accuracy *A_c_* by:

(12) $$A_{c}=N_{correct}/N_{batch},$$

where $N_{correct}$ and $N_{batch}$ denote the sample numbers of correct identification and current batch, respectively. We also use the accuracy defined in Formula (12) in Section 4.2.2. In Section 4.3, Section 4.4 and Section 4.5, we redefine accuracy *A_c_* considering both the data pool and the test set as follows:

(13) $$A_{c}=\left(N_{correct}^{1}+N_{correct}^{2}\right)/N_{batch},$$

where $N_{correct}^{1}$ and $N_{correct}^{2}$ are the numbers of the corrected recognized samples in the data pool and the test set, respectively.

We collect the accuracies from Batches 2 to 10 and provide the average values in the last column. In terms of the average accuracy, CC-LDA and CC-OSC have poor performance compared with other methodologies. The reason for this is that the decomposed drift-like component cannot keep up with the trend of actual drift. For the other methods, periodical correction is required, and we discover that the majority of AL methods have an excellent average accuracy (> 85%). In particular, AL-DC-ER (20) obtains the best results among all of the presented methodologies, with an accuracy of 93.06%. In terms of the IC type, TCTL+SEMI and DAELM-T achieved satisfactory rates of 87.60% and 85.62%, respectively. We infer that the occasional correction and sample label are the main reasons for these impressive outcomes. LFC-type methods maintain accuracies below 80% due to the lack of reliable labels. We also notice that the selective labelling in AL shows more flexibility than supervised learning does. For AL type methods, we can see that the AL-DC type methods (AL-DC-US and AL-DC-ER) provide more favorable results than AL-ACR under the same conditions. With respect to accuracies by batches, the AL-DC methods won 7 out of 9 comparisons. Furthermore, the recognition rates of Batch 10 decrease dramatically in all methods, which may be caused by the 5-month break between Batches 9 and 10.

#### 4.2.2. Short-Term Drift 

According to Setting 2, we summarize the recognition results of anti-drift methods in Table 4. With regard to the average accuracy, the CC approach generally lags behind the other types. DAELM-T has an accuracy of 85.10%, which is obviously higher than that of the CC and LFC methods. We attribute this to periodical drift correction with sample labels. The shortage of sample labels makes the LFC methods poorer than DAELM-T. The average accuracies of the AL methods are commonly higher than those of the other types. It is noticeable that the methods belonging to the AL-DC type again show greater recognition performance than AL-ACR. In particular, AL-DC-ER (20) achieves the highest average rate, at 93.74%, among all the methods. 

For the short-term drift scenarios presented, the AL-DC-type methods achieve the highest results in 8 out of 9 cases, and AL-ACR won the remaining one. Therefore, the AL-DC-type methods demonstrate excellent performance for short-term drift compensation.

Based on the results shown in Table 3 and Table 4, we can draw the following two conclusions: (1) AL-based drift correction is superior to any other type with respect to accuracy; and (2) AL-DC methods, especially AL-DC-ER, outweigh AL-ACR with respect to drift counteraction.

### 4.3. Parameter Sensitivity

**Figure 4:**
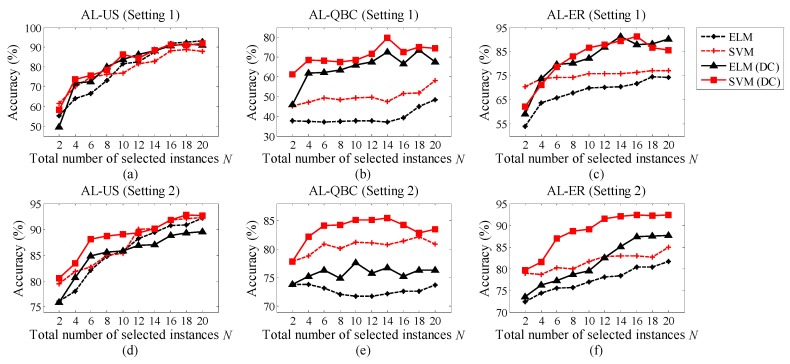
(**a**) Accuracy of AL-US-type methods in Setting 1; (**b**) accuracy of AL-QBC-type methods in Setting 1; (**c**) accuracy of AL-ER-type methods in Setting 1; (**d**) accuracy of AL-US-type methods in Setting 2; (**e**) accuracy of AL-QBC-type methods in Setting 2; (**f**) accuracy of AL-ER-type methods in Setting 2.

In this section, we intend to show the performance variation of the proposed AL-DC paradigm with the number of the selected instances *N*. As demonstrated in Figure 4a–f, we use the lines with black asterisks, red crosses, black triangles, and red squares to denote the accuracy of AL-ELM, AL-SVM, AL-DC-ELM, and AL-DC-SVM, respectively. Here, ELM and SVM indicate the classifier type used in the recognition and instance selection. The subplots in the first row represent the accuracy variations of the long-term scheme, while those in the second row are related to the short-term scheme. US, QBC, and ER strategies were adopted based on the subplots in the first, second and third columns, respectively. We discover that the AL methods on SVMs achieve better accuracy than the ELMs do. Moreover, the AL-DC methods are clearly superior to the traditional ALs when using the same classifier. When the number of selected instances *N* increases, the accuracy curves of AL-DC methods increase faster than the referenced ones. This confirms the excellent performance of the AL-DC type methods. Furthermore, the AL-DC methods often begin to increase their accuracies after *N* = 6. The reason for this is that the category of the drift dataset is equal to 6. For AL-DC, the training set with full categories is easier to form when *N* ≥ category number. After a rapid expansion around *N* = 6, the AL-DC curve enters a stable growth phase. In this phase, the overall trend of the curve is slightly upward with increasing *N*. Considering large *N* leads to extra computational and labor costs; we assume that the most favorable value of parameter *N* should be beyond and close to the category number. Of course, larger *N* is preferred if there is sufficient computational power, human resource and storage space.

### 4.4. Accuracy Increasing Process

**Figure 5:**
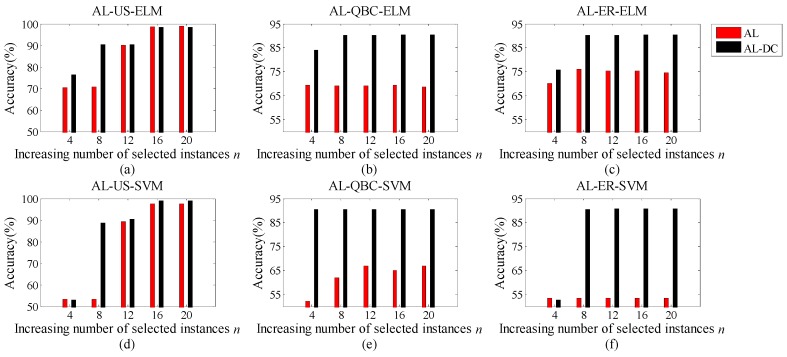
(**a**) Accuracy fluctuation on US with ELM; (**b**) accuracy fluctuation on QBC with ELM; (**c**) accuracy fluctuation on ER with ELM; (**d**) accuracy fluctuation on US with SVM; (**e**) accuracy fluctuation on QBC with SVM; (**f**) accuracy fluctuation on ER with SVM.

To explore the reason that AL-DC methodologies obtain excellent results, we show the accuracy increasing process for both the AL and AL-DC methodologies in this subsection. We choose a situation that existed in both Setting 1 and 2, that is, Batch 1 is used as the initial training set, while Batch 2 is treated as online drifted data. This means that the data pool and the testing data for the AL methods are all generated from Batch 2. As shown in Figure 5a–f, the heights of the red and black bars denote the accuracies of various AL and AL-DC methodologies, respectively. We set the maximum number of selected instances as *N* = 20 for one AL process, *n* (1 < *n* ≤ *N*) denotes the increasing number of selected instances during the AL process. Each subfigure describes the accuracy fluctuation with increasing *n* by a certain AL framework. The first to third columns in Figure 5 represent the results by using US, QBC, and ER strategies, respectively. The first line refers to the methods utilizing ELM as classifier, while the second line is based on SVM. We discovered two interesting findings: one is that the AL-DC-based methodologies achieve higher accuracy than traditional AL methods at the end of the AL process (*n* = *N*) in most cases. The other is that AL-DC-based methodologies have a faster convergence speed in all scenarios. In other words, they can obtain near-optimal results with smaller *n* (lower labelling cost) compared with the referenced methods. This is a favorable characteristic for online drift corrections considering the labelling cost. For the AL-DC methodologies, no matter which classifier or instance-selection strategy is used, as long as *n* exceeds 6, the recognition rates will be almost the same as the final value. Coincidentally, the category number of the dataset is 6, as well. We believe that this phenomenon is attributable to the DC strategy, which can select all kinds of samples if *n ≥* category number. A full-category training set would obviously promote the performance of the classifiers.

In terms of instance-selection strategy, the US strategy is more effective than the QBC and ER strategies under the AL-DC framework. All three strategies on AL-DC demonstrate a high speed of accuracy convergence. When the US strategy is adopted, the highest accuracy reaches around 99%. For classifiers, although ELM and SVM have different convergence speeds in some cases, they will eventually achieve almost the same recognition rate at the end of the AL process.

### 4.5. Execution Efficiency

When continuous drifted instances enter, the drift correction process switches to an online mode. Thus, shorter time, less computation, and less labelling for drift correction becomes crucial. To evaluate the efficiency of AL methods, we use LEI and define it as follows:

(14) LEI=α·A+(1−α)·ΔA,

where *A* is the accuracy before the last update, Δ*A* is the accuracy increment after the updating with the recent labelling instance. *a* is an adjustment parameter belonging to [0, 1], and we set *a* to 0.5 in this discussion. Higher *LEI* means that instances picked out by certain AL methods are beneficial to classification.

Table 5 shows the *LEI*s of AL and AL-DC with three different strategies in a long-term scenario when *N* ∈ [11, 20]. The *LEIs* of the AL-DC-US, AL-DC-QBC, and AL-DC-ER are higher than those of AL-US, AL-QBC, and AL-ER, respectively. From each row in the table, we discover that the value does not always increase as *N* increases. In other words, a peak may occur when *N* is in a certain range. In the long-term scenario, the optimal *N* values corresponding to the AL-US, AL-DC-US, AL-QBC, AL-DC-QBC, AL-ER, and AL-DC-ER methods are 18, 20, 19, 14, 20, and 16, respectively. For the short-term scenario, most of the *LEI*s in Table 6 are larger than the *LEI*s in the corresponding locations in Table 5, because the timescale of the short-term drift data is smaller than that in the long-term drift data. According to the relationship between *N* and *LEI* in Table 6, the optimal *N* corresponding to the AL-US, AL-DC-US, AL-QBC, AL-DC-QBC, AL-ER, and AL-DC-ER methods are 19, 18, 18, 11, 20, and 16 in the short-term scenario, respectively. From both Table 5 and Table 6, we conclude that AL-DC-type methodologies have larger *LEI* values than traditional AL methods. This proves that AL-DC is an efficient approach with respect to labelling cost and accuracy increasing speed.

## 5. Conclusions

To solve the drift problem of E-noses in an online situation, we proposed the AL-DC methodology, which selects instances using a specific strategy under drift effects. Initialization, clustering and instance selection are the three main stages of AL-DC, and these are compatible with common AL strategies, including US, QBC, and ER. We introduce a drift benchmark for E-noses to evaluate the proposed method with other state-of-art drift compensation approaches. The experimental results prove that AL-DC outmatches the other presented methods in long-term and short-term drift scenarios. Moreover, AL-DC is superior to other AL methods with the same selected instances, and has a faster convenience speed with respect to recognition rates. Additionally, the *LEI* value is discussed to assess the efficiency of AL methods. The results indicate that AL-DC is a low-consumption approach with respect to limited labelling time.

## Figures and Tables

**Table 1:** Brief of drift dataset.

Batch IDs	Recording Time	Sample Category	Dimension	Sample Number
Batch 1	Month 1–2	6	128	445
Batch 2	Month 3–4 and 8–10	6	128	1244
Batch 3	Month 11–13	5	128	1586
Batch 4	Month 14–15	5	128	161
Batch 5	Month 16	5	128	197
Batch 6	Month 17–20	6	128	2300
Batch 7	Month 21	6	128	3613
Batch 8	Month 22–23	6	128	294
Batch 9	Month 24 and 30	6	128	470
Batch 10	Month 36	6	128	3600

**Table 2:** Data allocation.

	Size of the Data Pool	Size of the Testing Samples
Batch 2	623	1244
Batch 3	794	1586
Batch 4	81	161
Batch 5	99	197
Batch 6	1151	2300
Batch 7	1807	3613
Batch 8	148	294
Batch 9	237	470
Batch 10	1740	3480

**Table 3:** Comparison of recognition accuracy in long-term drift (%).

Type	Method	Batch 2	Batch 3	Batch 4	Batch 5	Batch 6	Batch 7	Batch 8	Batch 9	Batch 10	Average
Component correction	CC-LDA	47.27	57.76	50.93	62.44	41.48	37.42	68.37	52.34	31.17	49.91
	CC-OSC	88.10	66.71	54.66	53.81	65.13	63.71	36.05	40.21	40.08	56.50
Instancecorrection	TCTL+SEMI	**96.95**	96.85	91.30	98.98	86.78	82.51	86.05	83.19	65.75	87.60
	DAELM-T (40)	83.52	96.34	88.20	**99.49**	78.43	80.93	87.42	**100.0**	56.25	85.62
Label free correction	SVM-comgfk	74.47	70.15	59.78	75.09	73.99	54.59	55.88	70.23	41.85	64.00
	ML-comgfk	80.25	74.99	78.79	67.41	77.82	71.68	49.96	50.79	53.79	67.28
	MFKS (20)	85.45	77.96	88.65	83.61	89.38	68.80	84.67	78.66	42.54	77.75
	DRCA	89.15	92.69	87.58	95.94	86.52	60.25	62.24	72.34	52.00	77.63
Active learning	AL-ACR (10)	90.03	83.67	92.55	98.48	78.09	74.54	92.86	74.89	62.16	83.03
	AL-ACR (20)	89.23	84.17	96.89	**99.49**	76.43	62.66	89.46	95.11	64.89	84.26
	AL-DC-US (10)	90.35	**99.18**	**100.00**	**99.49**	78.83	71.99	92.18	81.06	65.80	86.54
	AL-DC-QBC (10)	90.03	82.41	77.02	74.62	73.43	53.47	29.93	65.74	63.13	67.76
	AL-DC-ER (10)	90.68	98.42	96.27	98.48	90.30	81.87	90.14	75.96	62.67	87.20
	AL-DC-US (20)	90.59	97.79	**100.00**	**99.49**	79.30	**87.60**	**94.22**	96.60	68.59	90.47
	AL-DC-QBC (20)	90.27	82.47	62.73	75.63	73.65	74.81	84.35	63.19	67.21	74.93
	AL-DC-ER (20)	90.19	98.87	**100.00**	**99.49**	**96.35**	87.30	88.44	95.96	**80.98**	**93.06**

Note: the bold in each column of the table denotes the highest recognition accuracy to certain batch, the same below.

**Table 4:** Comparison of recognition accuracy in short-term drift (%).

Framework	Method	1→2	2→3	3→4	4→5	5→6	6→7	7→8	8→9	9→10	Average
Component correction	CC-LDA	47.27	46.72	70.81	85.28	48.87	75.15	77.21	62.77	30.25	60.48
Instance correction	DAELM-T (40)	83.52	96.41	81.36	96.45	85.13	80.49	85.71	**100.0**	56.81	85.10
Label free correction	SVM-comgfk	74.47	73.75	78.51	64.26	69.97	77.69	82.69	85.53	17.76	69.40
	ML-comgfk	80.25	98.55	84.89	89.85	75.53	91.17	61.22	95.53	39.56	79.62
	DRCA	89.15	98.11	95.03	69.54	50.87	78.94	65.99	84.04	36.31	74.22
Active learning	AL-ACR (10)	90.03	99.12	85.71	98.98	54.17	97.15	88.78	95.32	52.59	84.65
	AL-ACR (20)	89.87	98.87	85.73	98.98	76.27	97.23	94.22	87.44	**70.91**	88.84
	AL-DC-US (10)	**90.92**	99.31	98.76	98.98	96.96	98.42	94.56	87.66	50.34	90.66
	AL-DC-QBC (10)	90.59	99.12	98.76	98.48	75.48	93.16	92.18	78.72	51.58	86.45
	AL-DC-ER (10)	90.68	99.05	98.76	**99.49**	95.13	98.62	90.82	96.81	42.67	90.22
	AL-DC-US (20)	90.84	**99.87**	98.76	**99.49**	**98.65**	**99.47**	**94.90**	88.30	62.53	92.53
	AL-DC-QBC (20)	90.59	99.12	98.76	98.48	73.61	97.84	93.54	79.15	59.20	87.81
	AL-DC-ER (20)	90.59	99.31	**100.00**	**99.49**	98.09	98.84	**94.90**	98.30	64.14	**93.74**

**Table 5:** *LEI* (%, Setting 1).

Method	N = 11	N = 12	N = 13	N = 14	N = 15	N = 16	N = 17	N = 18	N = 19	N = 20	Optimal
AL-US	38.46	41.61	44.33	42.20	43.82	44.74	44.46	**45.00**	44.30	44.46	45.00 (*N* = 18)
AL-DC-US	43.83	43.15	44.22	45.05	44.01	46.43	43.80	46.15	45.79	**46.53**	46.53 (*N* = 20)
AL-QBC	24.59	25.03	24.32	23.88	25.74	25.86	25.30	26.08	**33.77**	29.20	33.77 (*N* = 19)
AL-DC-QBC	35.19	36.26	39.87	**40.53**	33.12	36.60	34.45	37.87	39.95	37.40	40.53 (*N* = 14)
AL-ER	38.40	38.44	38.44	38.43	38.62	38.61	38.54	38.93	38.97	**38.97**	38.97 (*N* = 20)
AL-DC-ER	43.61	44.80	44.12	45.64	44.87	**46.42**	44.32	43.89	44.92	43.42	46.42 (*N* = 16)

**Table 6:** *LEI* (%, Setting 2).

Method	N = 11	N = 12	N = 13	N = 14	N = 15	N = 16	N = 17	N = 18	N = 19	N = 20	Optimal
AL-US	43.07	45.57	45.59	45.55	46.27	46.37	46.44	46.42	**46.68**	46.53	46.68 (*N* = 19)
AL-DC-US	45.15	45.16	45.36	45.53	46.11	46.40	46.74	**46.82**	46.73	46.73	46.82 (*N* = 18)
AL-QBC	40.72	40.74	40.72	40.55	40.42	40.83	41.16	**41.25**	40.97	40.56	41.25 (*N* = 18)
AL-DC-QBC	**43.06**	42.93	42.84	43.01	42.46	42.33	42.34	41.57	41.93	41.89	43.06 (*N* = 11)
AL-ER	41.02	41.67	41.64	41.67	41.66	41.67	41.66	41.48	41.53	**42.66**	42.66 (*N* = 20)
AL-DC-ER	45.29	46.36	46.29	46.60	46.62	**46.63**	46.59	46.54	46.58	46.58	46.63 (*N* = 16)
